# Letrozole-Induced Pneumonitis Complicated by Acute Respiratory Distress Syndrome: A Case Report

**DOI:** 10.7759/cureus.108734

**Published:** 2026-05-12

**Authors:** Ameni Khaled, Charbel Mezeraany, Toboure Zanze, Ilef Alila, Kais Regaieg

**Affiliations:** 1 Intensive Care Unit, Groupement Hospitalier de Territoire Grand Paris Nord-Est, Montfermeil, FRA; 2 Department of Critical Care Medicine, Groupement Hospitalier de Territoire Grand Paris Nord-Est, Paris, FRA; 3 Department of Anesthesiology and Reanimation, Groupement Hospitalier de Territoire Grand Paris Nord-Est, Paris, FRA; 4 Intensive Care Unit, Groupement Hospitalier de Territoire Grand Paris Nord-Est, Paris, FRA

**Keywords:** ards, breast cancer, corticosteroids, drug-induced pneumonitis, letrozole

## Abstract

Letrozole is a commonly used aromatase inhibitor in the adjuvant treatment of hormone receptor-positive breast cancer and is generally well tolerated. Pulmonary toxicity is rare and poorly described. We report the case of a 74-year-old woman who developed progressive respiratory symptoms over two months, initially suggestive of infectious pneumonia, but with negative microbiological investigations and no response to multiple antibiotic therapies. Her condition worsened, leading to acute respiratory distress syndrome requiring invasive mechanical ventilation. A drug-induced pneumonitis related to letrozole was suspected. Discontinuation of the drug and initiation of corticosteroid therapy resulted in rapid clinical improvement. This case highlights a rare but potentially severe pulmonary adverse effect that should be considered in patients with unexplained pneumonia.

## Introduction

Third-generation aromatase inhibitors, including letrozole, play a central role in the adjuvant treatment of hormone receptor-positive breast cancer in postmenopausal women. By inhibiting the peripheral conversion of androgens into estrogens, they effectively suppress hormonal stimulation of tumor growth and significantly reduce recurrence risk and improve survival [1].

Letrozole is generally well tolerated, with adverse effects mainly related to estrogen deprivation, such as arthralgia, hot flashes, and bone loss [2]. In contrast, pulmonary toxicity associated with letrozole is extremely rare and remains poorly described.

While several anticancer agents are well known to cause pulmonary toxicity, aromatase inhibitors are rarely implicated. Drug-induced interstitial lung disease (DI-ILD) represents a heterogeneous group of pulmonary disorders with variable clinical and radiological presentations, making diagnosis particularly challenging in clinical practice [3]. Letrozole-induced pneumonitis remains exceptionally rare, with only a limited number of cases reported in the literature.

A few isolated cases of organizing pneumonia associated with letrozole have been reported, typically with favorable outcomes following drug discontinuation and corticosteroid therapy [4,5]. However, severe presentations, particularly those complicated by acute respiratory distress syndrome (ARDS), have not been clearly documented in the available literature and remain exceptional.

We report the case of a 74-year-old woman treated with letrozole who developed severe interstitial pneumonitis complicated by ARDS, with a favorable outcome after drug discontinuation and corticosteroid therapy.

## Case presentation

A 74-year-old woman was admitted for acute respiratory failure. She had a medical history of hypertension, type 2 diabetes mellitus, hypothyroidism, anxiety-depressive disorder, dyslipidemia, and left breast adenocarcinoma treated by lumpectomy followed by radiotherapy one year prior to this admission. She was in remission and had been receiving adjuvant hormonal therapy with letrozole (2.5 mg daily) for 10 months. Her usual medications included metformin, simvastatin, irbesartan, levothyroxine, sertraline, bromazepam, and letrozole. She was independent in daily activities, with no active smoking or alcohol consumption, no recent travel history, and no relevant environmental exposure.

Symptoms began approximately two months prior to admission, with progressive asthenia, worsening exertional dyspnea, persistent dry cough, and intermittent fever with night sweats. She presented to the emergency department due to worsening dyspnea.

On admission, she was alert, with blood pressure of 125/77 mmHg, heart rate of 70 beats/minute, oxygen saturation of 90% on room air, respiratory rate of 20 breaths/minute, and bilateral diffuse crackles on lung auscultation. She was afebrile. Laboratory findings showed a marked inflammatory response with leukocytosis and elevated inflammatory markers, while procalcitonin levels remained low (Table 1). 

**Table 1:** Laboratory results at admission A marked inflammatory response is seen with elevated C-reactive protein and leukocytosis, contrasting with low procalcitonin levels. ALT: alanine transaminase; AST: aspartate aminotransferase

Parameter	Patient Value	Reference Range	Interpretation
Hemoglobin	9.6 g/dL	12.0–16.0	Decreased
Leukocytes	13.0 G/L	4.0–10.0	Increased
Platelets	378 G/L	150–400	Normal
Sodium	139 mmol/L	136–145	Normal
Potassium	4.0 mmol/L	3.5–5.1	Normal
Chloride	101 mmol/L	98–107	Normal
Bicarbonate (HCO₃⁻)	28 mmol/L	22–26	Slightly increased
Creatinine	59 µmol/L	49–90	Normal
Urea	3.1 mmol/L	2.5–6.4	Normal
Total proteins	61 g/L	64–82	Slightly decreased
Creatine phosphokinase	77 U/L	30–190	Normal
AST	47 U/L	15–37	Slightly increased
ALT	50 U/L	13–56	Normal
Total bilirubin	11 µmol/L	3–17	Normal
C-reactive protein	299 mg/L	<5	Increased
Procalcitonin	0.13 ng/mL	<0.05	Slightly increased
Troponin (hs)	<10 ng/L	<14	Normal
Arterial pH	7.44	7.35–7.45	Normal
Lactate	1.7 mmol/L	0.5–1.6	Slightly increased

**Figure 1:**
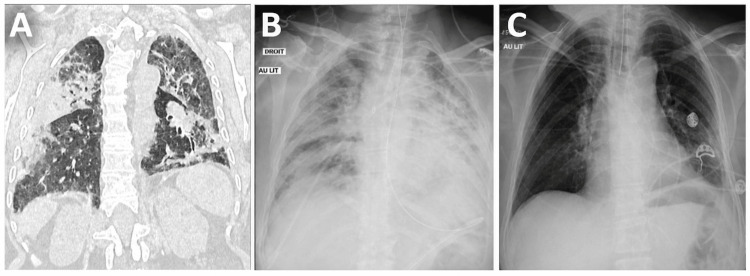
Imaging findings and disease evolution (A) Chest computed tomography (CT) showing bilateral multilobar consolidations with ground-glass opacities, predominantly in the left lung. (B) Chest radiograph demonstrating extensive bilateral alveolar opacities consistent with acute respiratory distress syndrome (ARDS). (C) Follow-up chest radiograph showing marked regression of pulmonary infiltrates after corticosteroid therapy and discontinuation of letrozole.

Electrocardiography showed a normal sinus rhythm without acute ischemic changes. Transthoracic echocardiography revealed preserved left ventricular systolic function without significant valvular abnormalities or evidence of cardiogenic pulmonary edema. Chest computed tomography (Figure 1A) revealed bilateral multilobar parenchymal consolidations, predominantly affecting the left lung, associated with diffuse ground-glass opacities and bronchial wall thickening, without evidence of pulmonary embolism. 

She was initially admitted to a medical ward and received oxygen therapy at 2 L/minute. Despite multiple courses of empirical antibiotics, including amoxicillin-clavulanate, cefotaxime plus spiramycin, and then piperacillin-tazobactam, her condition progressively worsened, with increasing oxygen requirements and no clinical or biological improvement. Initial microbiological investigations, including blood cultures, sputum culture, pneumococcal and Legionella urinary antigen tests, and respiratory viral polymerase chain reaction (PCR), were negative.

Two weeks later, because of worsening respiratory status with oxygen requirements rising to 10 L/minute, she was transferred to the intensive care unit. She rapidly developed acute respiratory distress syndrome requiring endotracheal intubation, invasive mechanical ventilation, neuromuscular blockade, and prone positioning. Chest radiograph (Figure 1B) performed at that time showed extensive bilateral alveolar opacities consistent with acute respiratory distress syndrome.

Extensive microbiological investigations, including repeated blood cultures, tracheal aspirates, extended respiratory PCR panels, atypical pathogens, mycobacteria, *Pneumocystis jirovecii*, herpes viruses, and aspergillosis, were all negative. No infectious pathogen was identified despite repeated testing.

Bronchoalveolar lavage (BAL) revealed a mixed inflammatory pattern without identification of infectious agents or malignant cells, supporting an inflammatory alveolar process. Autoimmune workup was negative. Given the history of intermittent fever and night sweats, tuberculosis was considered in the differential diagnosis. Acid-fast bacilli staining, mycobacterial cultures, and tuberculosis PCR performed on BAL were negative.

Given the prolonged exposure to letrozole, the subacute clinical course, the absence of microbiological documentation despite extensive investigations, the failure of successive antibiotic therapies, and the BAL findings, letrozole-induced pneumonitis was strongly suspected.

Letrozole was discontinued, and systemic corticosteroid therapy with intravenous methylprednisolone 80 mg/day was initiated, followed by progressive tapering over one month, with the dose reduced by half each week after clinical improvement. The patient required invasive mechanical ventilation for two weeks. The clinical course was rapidly favorable, with improvement in respiratory parameters within days, allowing progressive weaning from sedation and successful extubation.

Follow-up chest radiograph (Figure 1C) showed marked regression of pulmonary infiltrates after corticosteroid therapy and discontinuation of letrozole. The patient was subsequently weaned off oxygen therapy and transferred to a medical ward for further management. After multidisciplinary discussion, tamoxifen was considered as an alternative endocrine therapy.

## Discussion

Letrozole is a non-steroidal third-generation aromatase inhibitor widely used in the adjuvant treatment of hormone receptor-positive breast cancer in postmenopausal women [1]. It acts by competitively inhibiting aromatase (CYP19A1), the key enzyme involved in the peripheral conversion of androgens into estrogens, thereby inducing near-complete suppression of estrogen synthesis. Its oral bioavailability is high, and its elimination half-life is approximately two days, resulting in sustained systemic exposure [2]. Overall, letrozole is well tolerated, with adverse effects mainly related to estrogen deprivation. In contrast, pulmonary toxicity associated with letrozole is exceptional and probably underrecognized in routine practice.

Pulmonary toxicity related to letrozole is extremely rare, with only a limited number of published cases describing interstitial pneumonitis or organizing pneumonia. Our case highlights an unusually severe presentation complicated by ARDS, which remains exceptional. A first case reported in 2023 described letrozole-induced organizing pneumonia with favorable evolution after drug discontinuation [4]. More recently, a 2025 report also described organizing pneumonia associated with letrozole therapy, with improvement after treatment withdrawal [5]. These observations suggest that aromatase inhibitors may induce inflammatory lung injury with variable severity.

The pathophysiological mechanisms underlying this pulmonary toxicity remain poorly understood. The main hypothesis involves a delayed immune-mediated hypersensitivity reaction, supported by the subacute onset, inflammatory BAL findings, and favorable response to corticosteroids [3]. In addition, estrogen deprivation induced by letrozole may dysregulate pulmonary immune responses, since estrogens are known to modulate inflammation and tissue repair. Their reduction may promote excessive interstitial inflammation [6]. Finally, the rare, unpredictable, and non-dose-dependent nature of this toxicity suggests an idiosyncratic mechanism, possibly related to individual susceptibility [3].

The diagnosis of drug-induced interstitial lung disease remains a diagnosis of exclusion, based on converging clinical, radiological, and evolutionary arguments [3]. In our case, several features strongly supported this diagnosis: a compatible temporal relationship with letrozole exposure, subacute progression, absence of infectious documentation despite exhaustive microbiological investigations, failure of successive antibiotic regimens, and non-specific inflammatory BAL findings. The absence of evidence for autoimmune or neoplastic disease further reinforced this hypothesis. Finally, the rapid improvement after drug withdrawal and corticosteroid initiation strongly supported a drug-related cause.

The main differential diagnoses include infectious pneumonia, particularly opportunistic infections, radiation-induced lung injury, and neoplastic pulmonary involvement [6]. In our case, these diagnoses were reasonably excluded based on negative microbiological results, negative autoimmune investigations, and favorable evolution after the specific therapeutic strategy. Importantly, in our patient, the rapid response to corticosteroid therapy represents an additional argument supporting the diagnosis of drug-induced pneumonitis.

Management relies primarily on immediate discontinuation of the suspected drug, combined with systemic corticosteroid therapy in moderate to severe cases [7,8]. Clinical improvement is often rapid, as illustrated in our observation. Rechallenge with letrozole is not recommended because of the risk of recurrence.

## Conclusions

Letrozole-induced pneumonitis is a rare but potentially severe adverse effect that may progress to ARDS. Its presentation can mimic infectious pneumonia, leading to diagnostic delay and unnecessary antibiotic therapy. In patients receiving aromatase inhibitors with persistent respiratory symptoms and negative microbiological investigations, a drug-induced etiology should be considered. Early recognition, discontinuation of letrozole, and corticosteroid therapy are essential for favorable outcomes.

## References

[1] Goss JR, Elmore JG, Lessler DS (2003). Quality of health care delivered to adults in the United States. N Engl J Med.

[2] Bhatnagar AS (2007). The discovery and mechanism of action of letrozole. Breast Cancer Res Treat.

[3] Camus PH, Foucher P, Bonniaud PH, Ask K (2001). Drug-induced infiltrative lung disease. Eur Respir J.

[4] Bukkuri A, Mayo-Malasky H, Pillai M (2023). Letrozole-induced organizing pneumonia: a case report. Chest.

[5] Picado C, Soler N (2025). Organizing pneumonia caused by letrozole therapy for breast cancer: a case report. Arch Bronconeumol.

[6] Vegeto E, Benedusi V, Maggi A (2008). Estrogen anti-inflammatory activity in brain: a therapeutic opportunity for menopause and neurodegenerative diseases. Front Neuroendocrinol.

[7] Skeoch S, Weatherley N, Swift AJ (2018). Drug-induced interstitial lung disease: a systematic review. J Clin Med.

[8] Matsuno O (2012). Drug-induced interstitial lung disease: mechanisms and best diagnostic approaches. Respir Res.

